# Cutaneous Pyoderma Gangrenosum of the Hand

**Published:** 2018-02-05

**Authors:** Toni Mihailidis, David Izadi, Sameer Gujral

**Affiliations:** ^a^Department of Medicine, University of Bristol, Bristol, UK; ^b^Department of Plastic Surgery, Royal Devon & Exeter Hospital, Exeter, UK; ^c^Department of Plastic Surgery, Southmead Hospital, Bristol, UK

**Keywords:** Pyoderma gangrenosum, surgical debridement, contraindication, pathergy, immunosuppression

## DESCRIPTION

A 65-year-old man presented with a left nontraumatic thumb lesion. Inflammatory markers were raised and infection was initially diagnosed. Despite surgical debridements and intravenous antibiotics, wounds deteriorated, extending to his index finger. Microbiology and histology were nonspecific. A diagnosis of pyoderma gangrenosum (PG) was made and successful treatment with steroids was initiated.

## QUESTIONS

1. What is PG?
2. How is PG diagnosed?
3. What are the clinical and histological features of PG?
4. How is PG managed?

## DISCUSSION

Pyoderma gangrenosum is a rare, autoimmune, ulcerative, neutrophilic dermatosis of unknown aetiology. Initial presentation of PG is as small papules, which subsequently coalesce, break down, and undergo liquefactive necrosis to form ulcers.^1^ It has an estimated incidence of 3 to 10 cases per million worldwide and mainly affects adults, affecting men and women equally. Its peak onset is at 50 years of age.^2^ There are 5 clinical subtypes: ulcerative, peristomal, pustular, bullous, and vegetative. It is a rapidly progressive disease with irregular, erythematous undermined borders that can occur on any skin surface.^1^

Pyoderma gangrenosum is a diagnosis of exclusion and no laboratory test has been developed for its diagnosis.^3^ As illustrated by this case, PG is often not recognized immediately. This delay in diagnosis can have significant consequences.^1^ Although PG has a typical clinical appearance (discussed below), there are other conditions that present with similar features. Differential diagnoses include Behcet's, vasculitis, mixed cryoglobulinaemia, lupus, cutaneous infection such as ecthyma, blastomycosis, or herpes simplex, vascular or arterial ulceration, or cutaneous malignancy.^3^ An essential differential diagnosis of PG and possibility in our patient is Sweet syndrome: another neutrophilic dermatosis presenting with red papules and plaques, more commonly on the dorsum of the hand.^2^ Presently, PG lacks international diagnostic criteria, but some have been proposed, split into major and minor criteria. Major criteria include rapidly developing ulcers and the ruling-out of other ulcerative skin conditions. Minor criteria include pathergy (a nonspecific hypersensitivity reaction of skin to minimal trauma, leading to persistent ulceration at the site of surgical incision), systemic disease associated with PG, and more.^3^

Patients often present systemically unwell, with a fever.^1^ Lesions are painful, often severely so, and are disproportionate to the size of the lesion. Ulcers subsequently heal with cribriform scarring.^3^ In 50% to 70% of cases, PG presents with underlying inflammatory bowel disease, haematological malignancies, or rheumatologic disease.^2^ Extracutaneous manifestations may occur, including in the spleen, in the lungs as pulmonary nodules, or in the eyes as scleritis.^4^ Inflammatory markers, including C-reactive protein, erythrocyte sedimentation rate, and white blood cell count are raised. Furthermore, patients with PG may have raised autoantibodies (particularly p-ANCA) if inflammatory bowel disease is present. Histological appearance is contingent upon the subtype of PG as well as the time the skin biopsy is taken: early lesions show moderate lymphocytic infiltration with oedema and thrombosis. Infarction and abscesses occur in later ulceration, with antibodies such as IgM found in dermal blood vessels, suggesting an immunological pathogenesis.^5^ Clonal T-cell expansions have also been described in some PG lesions.^2^ However, these findings are not specific to PG. It is important to note that review by a dermopathologist is recommended over standard pathologic interpretation.

Surgery is contraindicated in PG: as in our patient, PG can persist and enlarge with debridement.^6^ Treatment is largely empirical in nature.^1^ PG should be treated according to local guidelines as there is currently no gold standard of treatment.^4^ Smaller lesions may be managed with topical steroid preparations or intra-lesional steroid injections (hence why when taking a biopsy from a suspected PG lesion, using an injectable corticosteroid is recommended to contain a possible pathergy reaction). Larger and multiple lesions may require long-term systemic immunosuppressive drugs: for example, either oral prednisolone 0.75 mg/kg/day in a single dose or ciclosporin 4 mg/kg/day in two divided doses. This can be adjusted to a maximum of 1 mg/kg/day for prednisolone and 5 mg/kg/day for cyclosporin.^7^ More recently, monoclonal antibodies targeting tumor necrosis factor α, such as infliximab, have been shown to be safe and effective treatments for PG.^8^ Management of underlying disease and pain management of lesions are also essential.^4^

## SUMMARY

As illustrated by this case, cutaneous PG of the hand is a diagnosis of exclusion. Though rare, an index of suspicion should be maintained as PG can present similarly to other conditions in the hand such as fulminating infection and malignancy, that would traditionally be managed surgically. Inappropriate surgical debridement of PG in the hand can, however, lead to persistent and worsening ulceration and poor outcomes, such as digital amputation, so should be avoided. Although international diagnostic criteria are yet to be established, prompt diagnosis is essential in avoiding significant clinical consequences.

## Figures and Tables

**Figure 1:**
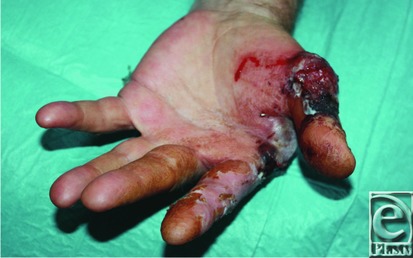
Palmar aspect of necrotizing lesion.

**Figure 2:**
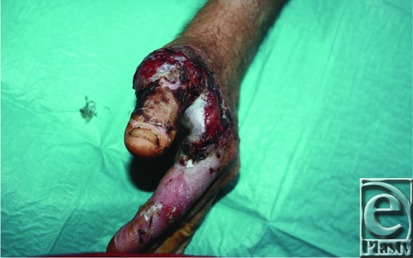
Lateral aspect of necrotizing lesion.

**Figure 3:**
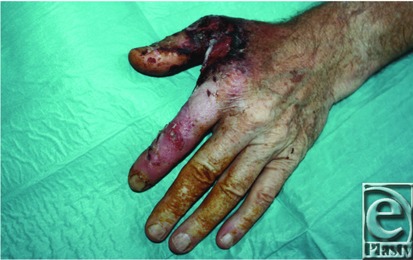
Dorsal aspect of necrotizing lesion.
